# A Note on Keramic Fillings

**Published:** 1905-03

**Authors:** William Rollins

**Affiliations:** Boston, Mass.


### A NOTE ON KERAMIC FILLINGS.

BY WILLIAM ROLLINS, BOSTON, MASS.

During the last few years several manufacturers, among whom may be mentioned Ash, Brewster, International Dental Manufacturing Company, Dr. N. S. Jenkins, and the S. S. White Company, have placed within reach of the profession excellent keramic materials for producing fillings for teeth by my method of fusing in foil matrices, and in consequence I have seen in the mouths of patients passing through Boston a considerable number of these fillings. Most of the work has been admirable, but some of the operations have been injudicious. In cases where the edge of the cavity was near or under the gum the work was not always well done, because the difficulties of getting a perfect foil matrix had been too great. As a result the layer of cement between the fillings and the edges of the cavities near the gum was too thick to be durable, and decay had begun. On this account a method of getting a suitable foil matrix from these cavities is given.

From the beginning, to save time, I endeavored to make keramic filling a laboratory method as far as possible, and soon perfected a means of making sharp impressions, which was described in my early papers and is the one used with the method to be described, only, instead of making an electrotype from the impression, a mould is obtained by using a zinc cement of the type described in my paper of 1879 and now known as oxyphosphate. This gives hard and perfect moulds, from the impressions of which there should be several. When the cement is hard the impression compound is removed. The part of the mould representing the gum is then ground away, giving free access to the cavity, thus facilitating the production of the foil matrix. The mould is then fastened by sealing-wax to the centre of a thin board twenty-five centimetres long, which serves as a rest for the hands while making the foil matrix, which is done from the zinc mould instead of from the tooth. The operation is simpler, and as many foil matrices can be made as are desirable, for, the work being done by an assistant, neither the dentist's nor the patient's time is taken. It is usually an advantage to have three fillings baked, as one is often a better color than the others. This method gives good impressions of the edges of cavities under the gum margins because the impression compound forces the gum away from the edges, leaving them sharply defined.

In keramic work I have employed for many years a binocular magnifying glass attached to the head and leaving both hands free. This was made by Heinrich Westien, of Mechlenburg. It has the advantage of allowing the work to be placed at the normal operating distance of about twenty-two centimetres, therefore all muscular movements seem normal. It is of especial value in manipulating the foil matrices and in baking, as it protects the eyes from the heat and allows the operation to be minutely watched during the whole fusion. For keramic material that fuses in gold matrices I have used, since its introduction some years ago, Mitchell's little furnace, which weighs only a hundred and seventy grammes. If the foil matrix is placed two centimetres from the open mouth of the muffle, fusion is perfect and the work can be constantly observed.

